# Chloroquine Potentiates the Chemotherapeutic Effect of Carboplatin and ATR/Chk1 Inhibitors by Increasing the Replication Stress

**DOI:** 10.3390/ijms27020856

**Published:** 2026-01-15

**Authors:** Maria Zamkova, Nadezhda Persiyantseva, Svetlana Vikhrova, Dmitriy Kazansky

**Affiliations:** 1 Institute of Gene Biology, Russian Academy of Sciences, 119334 Moscow, Russia; s_v00@bk.ru; 2 Blokhin National Medical Research Center of Oncology, 115478 Moscow, Russia; nadushka99@gmail.com (N.P.); kazansky1@yandex.ru (D.K.)

**Keywords:** chloroquine, carboplatin, ATR inhibitor, Chk1 inhibitor, replication stress, chemotherapy, cell cycle

## Abstract

Lysosomal inhibition by different agents like chloroquine and bafilomycin A is known to sensitize some tumor cells to chemotherapeutic drugs. The mechanism and signaling pathways are still under investigation. We showed that chloroquine sensitized tumor cells (MCF7, SKBR3, HCT116) to drugs (carboplatin, cisplatin) treatment. Treatment with the combination of platinum drugs and chloroquine resulted in the increased rate of apoptosis compared with single agent treatment. Moreover, we demonstrated the inhibition of the resumption of cell proliferation after cell cycle arrest induced by drugs treatment. Cells treated with the combination of carboplatin (or cisplatin) and chloroquine demonstrated the significant increase in Chk1 protein phosphorylation (Ser345), which together with S-phase increase indicated the induction of replication stress compared to cells treated with carboplatin (or cisplatin) alone. The rescue experiment performed by supplementation the combination of carboplatin and chloroquine with deoxyribonucleotides (dNTPs) demonstrated the reverse of inhibition of cells’ re-proliferation after cell cycle arrest caused by this combination of drugs. Treatment with carboplatin and ATR inhibitor (ceralasertib) greatly increased the level of phospho-Chk1 and induced the replication stress, which is consistent with previous studies. Supplementation of the above drug combination with chloroquine further increased Chk1 phosphorylation and decreased the number of cells able to re-proliferate after the induced stress. Here, we also demonstrated that dNTPs’ supplementation reversed the effect of chloroquine. Similar results were obtained with the combination of carboplatin and Chk1 inhibitor (prexasertib). It was also demonstrated that chloroquine could potentiate the effect of single agent treatment of tumor cells with ATRi/Chk1i in MCF7 cells. Here, we proposed a novel explanation for the chloroquine ability to potentiate the effect of chemotherapy. The results clearly demonstrated that stress induced by chloroquine is due to its ability to increase the replication stress and to reduce the availability of nucleotides.

## 1. Introduction

Combination chemotherapy is a promising and more effective strategy to treat cancer as it has benefits to conventional monotherapy. First, treatment with two or more therapeutic agents reduces the toxicity as they target different signaling pathways [1,2]. Common chemotherapeutic drugs used in tumor treatment target actively proliferating cells and can destruct healthy cells as well. Drugs combination allows us to reduce the therapeutic dosage as they usually work in a synergistic manner [3]. Second, combination chemotherapy helps to overcome drug resistance as fewer cycles of treatment are needed to achieve the chemotherapeutic effect [4]. Third, cancer stem cells are not eliminated by monotherapy, while combination treatment with agents that target CSC is more successful [5]. Finally, combination therapy improved treatment efficiency as drugs acted through different mechanisms, thus reducing the number of cancer cells able to survive, which led to a better chemotherapeutic response [6].

Platinum drugs are common drugs for various malignant tumors treatment including breast cancer, ovarian cancer, colorectal cancer, and others [7]. Despite their central role in cancer treatment, long-term administration leads to severe adverse effects, including nephrotoxicity (more common for cisplatin), thrombocytopenia caused by myelosuppression (carboplatin), dosage-limiting nausea and vomiting and others [7]. Combination therapy (or multiple-low-dose therapy (MLD)) seems an attractive strategy not only in overcoming drug resistance development but also in reducing the toxicities as the lower therapeutic drugs doses are needed.

Genotoxic chemotherapy induces replication stress that is a consequence of dysregulation of fork progression. Tumor cells respond by activation of pro-survival pathways, including DNA repair pathways that promote the restart of stalled forks [8]. Targeting proteins involved in replication stress response together with conventional chemotherapy is a promising approach as their inhibition abrogates stress response activation and thus results in a more effective killing of tumor cells. The targets include ATR, Chk1, Wee1, PARP1 and others [9]. Cells treated with such drugs combination are supposed not to be arrested after exposure to the agents but rather to continue proliferation that would lead to the mitotic catastrophe [10]. Prexasertib, a Chk1 inhibitor, showed good antitumor activity in preclinical animals studies [11] but failed in phase I/III clinical trial and has serious side effects [12,13]. Ceralasertib, ATR inhibitor, is better tolerated and has clinical benefits. However, the schedule and doses are needed to be optimized to reach the therapeutic effect—both prolong treatment and dose increase caused adverse effects [14].

Lysosomal inhibition together with conventional chemotherapy is another strategy to increase the therapeutic effect. Chloroquine (CQ) is one of the well-known autophagy inhibitors. Originally, it was discovered as an antimalarial agent in 1934 [15]. Later, its antitumor activity was described and CQ was proposed both as single agent and as an adjuvant in combination with various genotoxic drugs for cancer treatment. CQ is an attractive agent because of its low cost and oral bioavailability [16,17]. Its chemotherapeutic effect was shown for hematological and solid tumors [18] both in in vitro and in vivo studies. Despite this, the results obtained in clinical trials are not so successful—“partial response” has been reported, several trials could not reveal any changes in overall survival rates [17]. The mechanisms of CQ action are controversial and, as indicated in several studies, are independent of autophagy [19].

In general, CQ is a safe drug with low toxicity when used in recommended therapeutic doses. Gastrointestinal toxicities can occur when it is used in the short term, including nausea, vomiting and diarrhea. Long-term use and/or overdose can lead to retinopathy, cardiotoxicity and neurotoxicity. But, most of these toxicities are reversible if the treatment is stopped. Reducing the CQ dose significantly decreases its possible adverse effects [20,21]. Note that as CQ has a narrow therapeutic window and does not show good efficiency when used as a single chemotherapeutic agent [22] it is a priority to use it in combination with gentotoxic drugs in order to achieve the maximum chemotherapeutic efficiency along with side effects minimization due to low dose treatment. The reduced concentration of agents in such combination treatment can be used because of the presence of a synergistic effect between the drugs. So, it seems important to determine the appropriate drugs whose efficiency can be increased when combined with CQ.

Previous studies demonstrated that CQ acting as a single agent could enhance replication stress by decreasing nucleotides availability and thus impairs the DNA repair, which could result in replication fork collapse and mitotic catastrophe [23]. Here, we reported that CQ potentiated the chemotherapeutic effect of platinum-based drugs, inducing cell death and inhibiting the ability of tumor cells to re-proliferate after treatment. Supplementation with CQ increased replication stress as indicated by the elevated level of phospho-Chk1 (Ser345) and number of cells arrested in G1/S phase. Moreover, CQ potentiated the effect of replication stress response (RSR) inhibitors, ATRi and Chk1i, used both as adjuvants to carboplatin and as single agents. Thus, we found that low-dose CQ supplementation could reduce the chemotherapeutic doses of carboplatin and also ATRi and Chki used for cancer treatment and so makes the treatment less toxic without losing the therapeutic effect.

## 2. Results

### 2.1. CQ Inhibited the Re-Proliferation Potential of Breast Tumor Cells After Carboplatin Treatment

**Figure 1:**
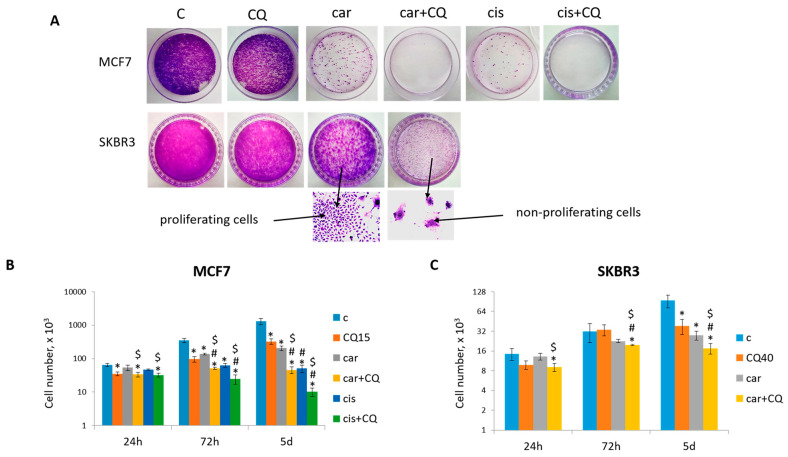
CQ potentiated the chemotherapeutic effect of platinum-based drugs. (**A**) The ability of MCF7 and SKBR3 cells to re-proliferate after drugs treatment. Cells were exposed to carboplatin (or cisplatin where indicated) and to the combination of carboplatin (cisplatin) and CQ for 24 h; next day drug-free medium was replenished, cells were re-seeded at low density and allowed to form colonies, which were stained with crystal violet 10 days after. The experiments were repeated at least three times; the representative experiments are shown. *Bottom*—the representative fields of the plates are shown at magnitude ×40 for visualization of proliferating and non-proliferating cells. (**B**,**C**) Cell growth assay. 3 × 10^4^ MCF7 cells and 7 × 10^3^ SKBR3 cells were seeded at day 0. Drugs were applied at day 1; next day drug-free media were replenished (24 h). The cells were counted in three time points (24 h, 72 h and 5 d). The experiments were repeated in two replicates three times. The averages ± st. dev. are shown. * *p* < 0.01 compared with non-treated cells; ^#^ *p* < 0.01 compared to CQ-treated cells; ^$^ *p* < 0.01 two-drug-treated cells compared with single drug (carboplatin or cisplatin)-treated cells. C—control (non-treated cells), CQ—chloroquine, car—carboplatin, cis—cisplatin.

The negative consequence of low-dose chemotherapy is cancer recurrence due to the ability of some tumor cells to escape from drug-induced apoptosis following the cell cycle arrest and beginning to re-proliferate. The main idea of our study was to evaluate the ability of CQ not only to increase cancer cell death when treated in combination with genotoxic drug but also to inhibit the ability of some cells to re-start proliferation after drug treatment. So, at first we analyzed the ability of breast tumor cells to form colonies after chemotherapy. As shown in Figure 1A, MCF7 cells treated with 50 µM carboplatin were able to re-proliferate and formed significant number of colonies. However, supplementation with 15 µM CQ almost completely inhibited the re-proliferation potential of the cells. Using another breast tumor cell line, SKBR3, we also demonstrated that 40 µM CQ potentiated the effect of 50 µM carboplatin—cells treated with drugs combination showed the reduced ability to re-proliferate after cell cycle arrest (Figure 1A).

The concentrations of drugs were determined using MTT test (Supplementary Figure S1A) and colony formation assay (Supplementary Figure S1B,C). As we proposed that CQ would potentiate the chemotherapeutic effect of carboplatin and thus allowed us to reduce its treatment dose, we tested tumor cells treated with low-dose carboplatin (between IC10 and IC30) for their ability to re-proliferate after treatment. Finally, we chose 50 µM carboplatin (see Figure 1A) as the lower concentration, leading to robust cells’ re-proliferation, and the higher dose resulted in significant inhibition of re-proliferation (Supplementary Figure S1B,C). This is a common concentration used in in vitro studies [24]. Evaluation of the ability of low-dose CQ (20–40 µM) [25] to inhibit the re-proliferation of tumor cells after treatment showed that its “best” concentration was 15 µM for MCF7 and 40 µM for SKBR3 cells (Supplementary Figure S1B,C).

Treatment MCF7 and SKBR3 cells with the combination of carboplatin and CQ resulted in significant cell growth inhibition compared with single agents’ treatment and non-treated cells (Figure 1B,C).

**Figure 2:**
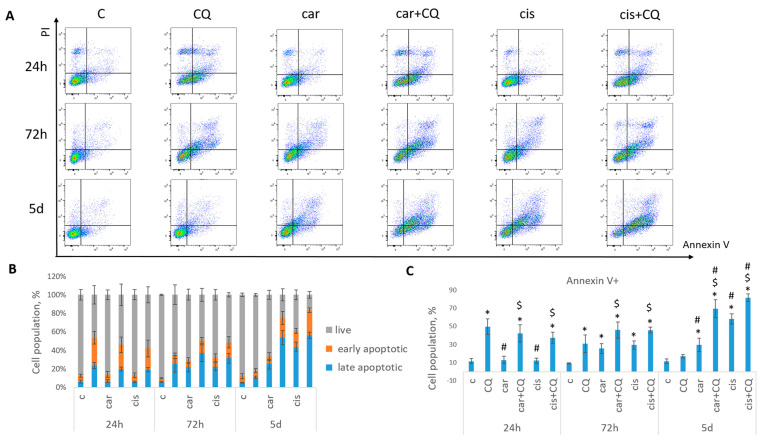
Synergistic effect of CQ and carboplatin on MCF7 cells death. Cells were treated with carboplatin (or cisplatin where indicated) and with the combination of carboplatin (cisplatin) and CQ for 24 h; next day drug-free medium was replenished. Annexin V/PI staining was performed in three time points (24 h, 72 h and 5 d). (**A**) The representative flow cytometry images are shown for each time point. (**B**) Histogram demonstrated the percentage of live, early and late apoptotic cells in each experimental group. (**C**) Cell populations stained positive for Annexin V are shown. Experiments were repeated at least three times. The averages ± st. dev. are shown. * *p* < 0.01 compared with non-treated cells; ^#^ *p* < 0.01 compared to CQ-treated cells; ^$^ *p* < 0.01 two-drug-treated cells compared with single drug (carboplatin or cisplatin)-treated cells. C—control (non-treated cells), CQ—chloroquine, car—carboplatin, cis—cisplatin.

**Figure 3:**
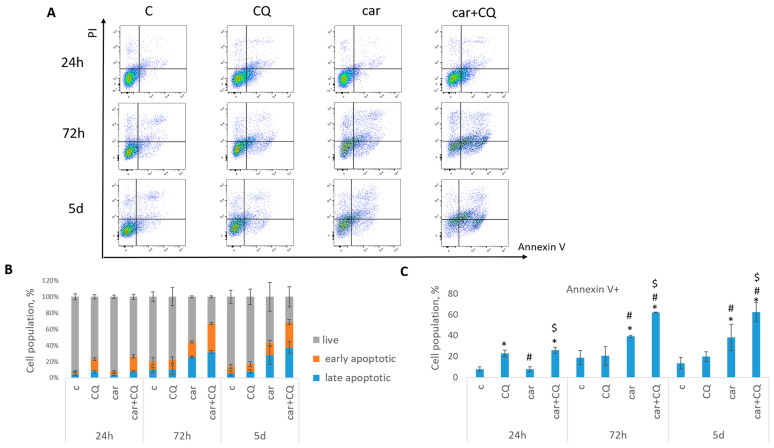
Synergistic effect of CQ and carboplatin on SKBR3 cells death. Cells were treated with carboplatin and with the combination of carboplatin and CQ for 24 h; next day drug-free medium was replenished. Annexin V/PI staining was performed in three time points (24 h, 72 h and 5 d). (**A**) The representative flow cytometry images are shown for each time point. (**B**) Histogram demonstrated the percentage of live, early and late apoptotic cells in each experimental group. (**C**) Cell populations stained positive for annexin V are shown. Experiments were repeated at least three times. The averages ± st. dev. are shown. * *p* < 0.01 compared with non-treated cells; ^#^ *p* < 0.01 compared to CQ-treated cells; ^$^ *p* < 0.01 two-drug-treated cells compared with single drug (carboplatin or cisplatin)-treated cells. C—control (non-treated cells), CQ—chloroquine, car—carboplatin.

Annexin V/PI staining was performed to detect the cell death phases (early and late apoptotic). It was shown that treatment with drugs combination led to the increased rate of apoptosis in 72 h and 5 d post treatment in both cell lines (MCF7 Figure 2, SKBR3 Figure 3) compared with control, CQ- and carboplatin-treated cells. Single CQ treatment resulted in significant increase in cell apoptosis in the first 72 h (MCF7) or 24 h (SKBR3) compared with non-treated cells. However, at day 5 (MCF7) or 72 h (SKBR3), the rate of apoptosis became comparable between these two groups.

Similar results were obtained using another platinum-based drug—cisplatin. Here, we also found that MCF7 cells treated with drugs combination (cisplatin + CQ) demonstrated significant inhibition of cells ability to re-proliferate post treatment, reduced cell growth (Figure 1) and enhanced rate of apoptosis in 72 h with its continued increase at day 5 post treatment (Figure 2).

Taken together, the data supported that CQ played the anti-tumor role in the combination with platinum drugs by increasing their cytotoxicity and abrogating the re-proliferation potential of tumor cells post treatment. Combination treatment allowed using the lower dosages of genotoxic drugs in order to achieve the maximum effect with toxicity reduction.

### 2.2. Combination of Carboplatin and CQ Enhanced Replication Stress

Carboplatin is known to induce the replication stress which is characterized by the increase in cells number arrested in S/G2 phase and the increase in protein Chk1 phosphorylation level (Ser345) [24,26], a known replication stress marker [23,27].

As shown earlier, lysosomal inhibition could also induce replication stress [23]. We hypothesized that the effects observed in our experiments with cells treated with the combination of chemotherapeutic drug and CQ were also due to CQ ability to enhance replication stress.

**Figure 4:**
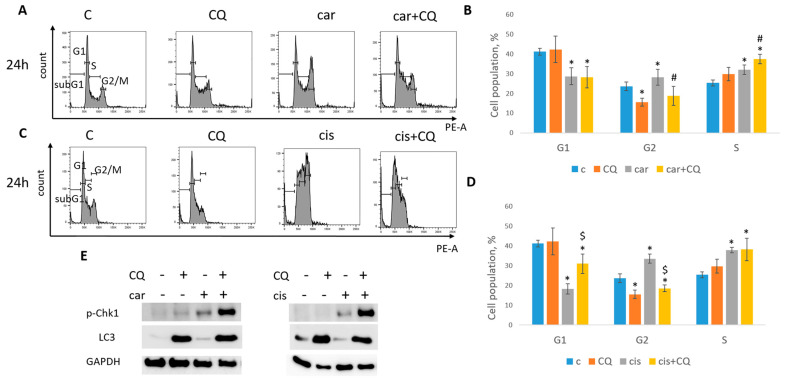
CQ enhanced replication stress in MCF7 cells. (**A**) Cell cycle analysis by flow cytometry. Cells were treated with carboplatin and combination of carboplatin and CQ. The representative images with cell cycle phases distribution are shown for each experimental group. (**B**) Histogram represents the percentage of cells in G1, S and G2/M cell cycle phases. (**C**) Cells were treated with cisplatin and combination of cisplatin and CQ. The representative images with cell cycle phase distribution are shown for each experimental group. (**D**) Histogram represents the percentage of cells in G1, S and G2/M cell cycle phases. Experiments were repeated at least three times. The averages ± st. dev. are shown. * *p* < 0.01 compared with non-treated cells; ^#^ *p* < 0.05 two-drug-treated cells compared with single drug (carboplatin)-treated cells; ^$^ *p* < 0.01 two-drug-treated cells compared with single drug (cisplatin)-treated cells. (**E**) Western blot analysis of replication stress marker p-Chk1 (Ser345) and autophagy marker LC3A/B. Experiments were repeated three times. GAPDH used as a loading control. C—control (non-treated cells), CQ—chloroquine, car—carboplatin.

**Figure 5:**
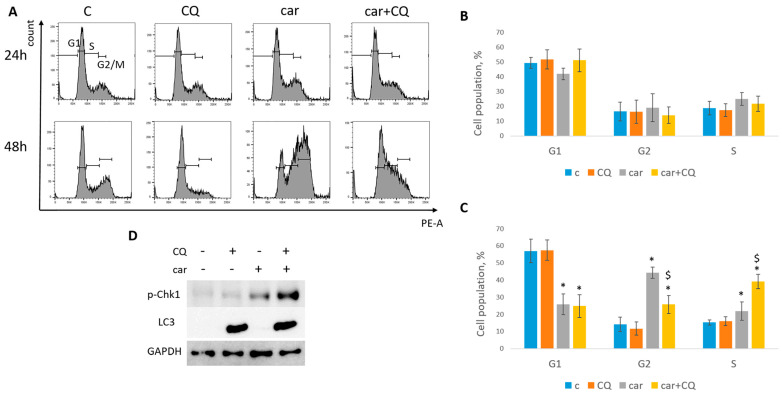
CQ enhanced replication stress in SKBR3 cells. (**A**) Cell cycle analysis by flow cytometry. Cells were treated with carboplatin and combination of carboplatin and CQ for 24 h. The representative images with cell cycle phase distribution 24 h (up) and 48 h (bottom) post treatment are shown for each experimental group. (**B**,**C**) Histogram represents the percentage of cells in G1, S and G2/M cell cycle phases 24 h (**B**) and 48 h (**C**) post treatment. Experiments were repeated at least three times. The averages ± st. dev. are shown. * *p* < 0.01 compared with non-treated cells; ^$^ *p* < 0.01 two-drug-treated cells compared with single drug (carboplatin)-treated cells. (**D**) Western blot analysis of replication stress marker p-Chk1 (Ser345) and autophagy marker LC3A/B. Experiments were repeated three times. GAPDH used as a loading control. C—control (non-treated cells), CQ—chloroquine, car—carboplatin.

At 24 h post drugs treatment, cell cycle analysis revealed that MCF7 cells were mostly arrested in S phase when treated with carboplatin together with chloroquine, while using single drug caused the S/G2 arrest (Figure 4A,B). At the same time point we could not detect the induction of neither G1/S nor G2 phase in SKBR3 cells (Figure 5A,B). However, cell cycle analysis at 48 h post treatment revealed robust induction of S phase in cells treated with combination of carboplatin and CQ. Single carboplatin treatment resulted in cell cycle arrest mostly in G2 phase at 48 h time point (Figure 5A,C). At 48 h post drugs treatment, MCF7 cells treated with both carboplatin and combination of carboplatin and CQ were predominantly arrested in G2/M phase and we also observed the increase in subG1 phase in combination-treated cells, which was consistent with annexin V/PI staining results (Supplementary Figure S2A,B).

MCF7 cells treated with both cisplatin and combination of cisplatin and CQ demonstrated the significant induction of S phase (Figure 4C,D). However, combination-treated cells also led to the increase in G1 phase, while cisplatin-treated cells to G2/M phase.

Based on this data, we concluded that replication stress was induced at different time points in MCF7 and SKBR3 cell lines. So, further experiments were performed at 24 h (post treatment) for MCF7 and 48 h for SKBR3 cells.

The replication stress induction is characterized by a robust increase in protein Chk1 phosphorylation at serine 345 [27]. Analysis of Chk1 phosphorylation (Ser345) level revealed its significant induction in cells (MCF7, SKBR3) treated with combination of carboplatin and CQ (Figure 4E and Figure 5D), which corresponded to the S phase increase. Autophagy marker, LC3A/B, served as a control to ensure that CQ actually acted as a lysosomal inhibitor. Similar results were obtained with the combination of cisplatin and CQ (MCF7)—a significant induction of Chk1 phosphorylation (Figure 4E).

Taken together, our results clearly demonstrated the ability of CQ to increase the replication stress caused by carboplatin/cisplatin treatment.

### 2.3. The Effect of Lysosomal Inhibition Was Not Restricted to Breast Cancer Cell Lines and CQ

To demonstrate that the effect of chloroquine in combination with carboplatin was not restricted to breast cancer cells, we treated CQ cells of different origins with 80 µM carboplatin and 40 µM—HCT116 (drugs concentrations were selected as it was described for breast tumor cell lines (Supplementary Figure S1)). The results were similar to previously described. Cell growth assay demonstrated the inhibition of cellular proliferation post drugs treatment both in carboplatin- and combination-treated groups compared to non-treated cells, with more pronounced inhibition in carboplatin + CQ-treated group (Supplementary Figure S2A). The evaluation of the ability of some cells to resume their proliferation post drugs treatment revealed its complete inhibition in combination-treated group (Supplementary Figures S1D and S2A).

The rate of apoptosis was determined using annexin V/PI staining protocol. Treatment with carboplatin and CQ resulted in the increased apoptotic rate beginning 24 h post treatment compared to control and carboplatin-treated groups (Supplementary Figure S2C–E). Single CQ treatment also caused cell death during the first 72 h post treatment, but at day 5, the rate of apoptosis was significantly decreased.

Cell cycle analysis did not reveal S phase induction in drugs combination-treated group 24 h post treatment. At this time point, single carboplatin treatment induced S phase arrest, while single CQ and combination treatment induced G1 phase (Supplementary Figure S3C). At 48 h post treatment, approximately half of the cell populations were in subG1 phase in these two groups, which was consistent with annexin V/PI staining results (Supplementary Figure S4A,B). The rest of the live cells were supposed to be those cells that in perspective would resume their proliferation. So, we gated this population and analyzed cell cycle phase distribution in it (Supplementary Figure S4C). As expected, drugs combination treatment as well as single CQ treatment significantly induced S phase arrest compared to single carboplatin treatment. At this time point, most of the cells treated with carboplatin alone were in G2/M cell cycle phase.

Next, we analyzed the level of replication stress marker—p-Chk1 (Ser345). Consistent with the cell cycle results, its level was induced in carboplatin-treated cells 24 h post treatment and in combination-treated cells 48 h post treatment—the same time points that were characterized by S phase induction. Moreover, carboplatin + CQ caused stronger induction of Chk1 phosphorylation than carboplatin alone (Supplementary Figure S4E).

To evaluate whether the effect was specific for CQ or could be reproduced with another lysosomal inhibitor, we performed the experiments described above using NH4Cl, another well-known lysosomal inhibitor [28]. Combination treatment with carboplatin and NH4Cl significantly inhibited the ability of arrested cells to resume their proliferation (Supplementary Figure S5A). Cell cycle analysis revealed the induction of G1/S phase when combination was applied, while single carboplatin treatment resulted in S/G2 increase (Supplementary Figure S5B,C). We also detected the significant increase in the protein level of replication stress marker, p-Chk1 (Ser345) (Supplementary Figure S5D).

Taken together, our data demonstrated that the replication stress increase and inhibition of tumor cells’ escape post treatment took place in different cell lines and were not a unique feature of CQ but rather due to its ability to inhibit lysosomal functions.

### 2.4. Exogenous Nucleotides Reversed the Ability of Combination to Inhibit the Resumption of Cells Proliferation

**Figure 6:**
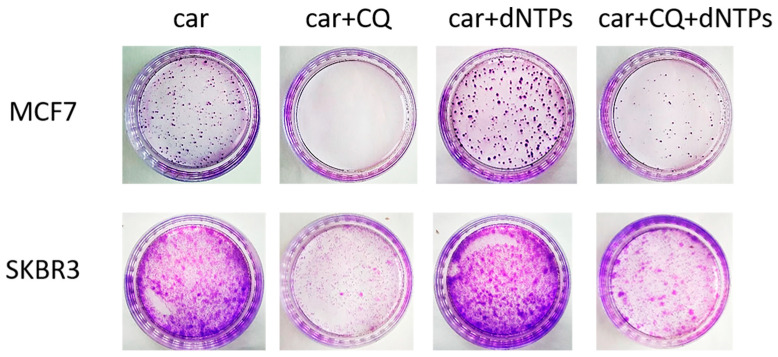
Supplementation with dNTPs reversed the ability of carboplatin + CQ to inhibit the resumption of tumor cells’ (MCF7 and SKBR3) proliferation post treatment. Cells were exposed to carboplatin and to the combination of carboplatin and CQ with and without dNTPs (20 µM [30]) for 24 h; next day drug-free medium was replenished, cells were re-seeded at low density and allowed to form colonies, which were stained with crystal violet 10 days after. The experiments were repeated at least three times; the representative experiments are shown. CQ—chloroquine, car—carboplatin.

Insufficiency of replication factors, including deoxyribonucleotides (dNTPs), is a known cause of stalled replication forks and replication stress [29]. We hypothesized that the induction of replication stress by CQ could be due to its ability to inhibit de novo nucleotide biosynthesis. To test this hypothesis, we performed the rescue experiment by administration of dNTPs to the combination of drugs (carboplatin + CQ). Supplementation with nucleotides partly reversed CQ effect. The number of colonies formed post drugs treatment was significantly higher in the presence of dNTPs in experimental groups treated with carboplatin and CQ both in MCF7 and SKBR3 cell lines (Figure 6).

### 2.5. CQ Potentiated the Effect of RSR Inhibitors

**Figure 7:**
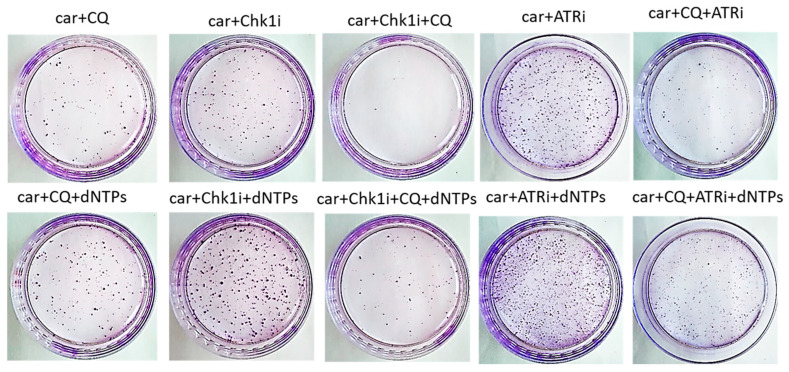
The ability of MCF7 cells to re-proliferate after drugs treatment. Cells were exposed to 50 µM carboplatin + ATRi (0.25 µM)/Chk1i (5 nM) and to the combination of carboplatin + ATRi/Chk1i + CQ (5 µM) for 24 h. Next day drug-free medium was replenished, cells were re-seeded at low density and allowed to form colonies, which were stained with crystal violet 10 days after. The experiments were repeated at least three times; the representative experiment is shown. CQ—chloroquine, car—carboplatin, ATRi—ATR inhibitor (ceralasertib), Chk1i—Chk1 inhibitor (prexasertib), dNTPs—deoxyribonucleotides.

**Figure 8:**
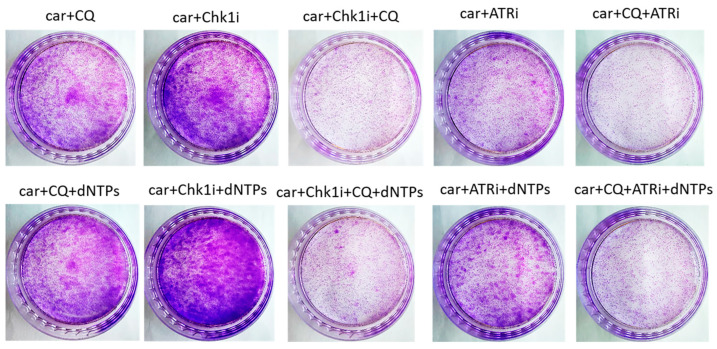
The ability of SKBR3 cells to re-proliferate after drugs treatment. Cells were exposed to 50 µM carboplatin + ATRi (0.25 µM)/Chk1i (1 nM) and to the combination of carboplatin + ATRi/Chk1i + CQ (20 µM) for 24 h. Next day drug-free medium was replenished, cells were re-seeded at low density and allowed to form colonies, which were stained with crystal violet 10 days after. The experiments were repeated at least three times; the representative experiment is shown. CQ—chloroquine, car—carboplatin, ATRi—ATR inhibitor (ceralasertib), Chk1i—Chk1 inhibitor (prexasertib), dNTPs—deoxyribonucleotides.

**Figure 9:**
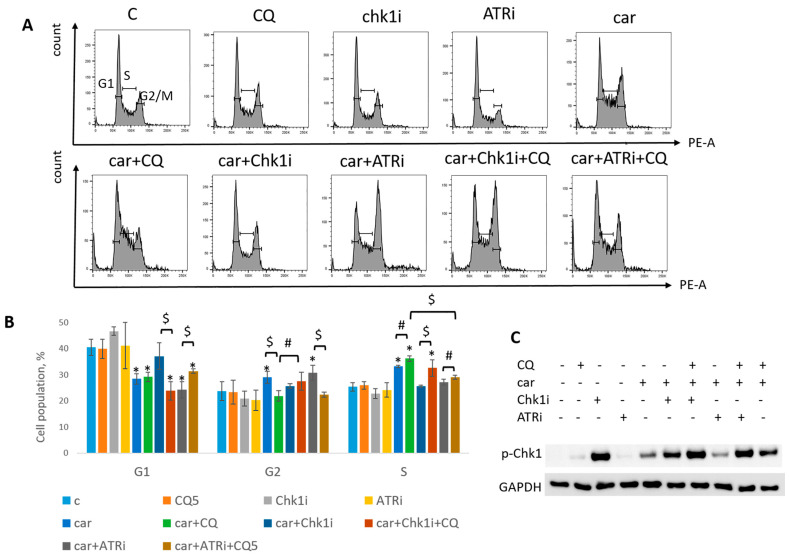
CQ potentiated the chemotherapeutic effect of carboplatin and RSR inhibitors in MCF7 cells. (**A**) Cell cycle analysis by flow cytometry. Cells were treated with 50 µM carboplatin + ATRi (0.25 µM)/Chk1i (5 nM) and combination of carboplatin + ATRi/Chk1i + CQ (5 µM). The representative images with cell cycle phase distribution are shown for each experimental group. (**B**) Histogram represents the percentage of cells in G1, S and G2/M cell cycle phases. The averages ± st. dev. are shown. * *p* < 0.01 compared with non-treated cells; ^$^ *p* < 0.01; ^#^ *p* < 0.05. (**C**) Western blot analysis of replication stress marker p-Chk1 (Ser345). GAPDH used as a loading control. Experiments were repeated three times. C—control (non-treated cells), CQ—chloroquine, car—carboplatin, ATRi—ATR inhibitor (ceralasertib), Chk1i—Chk1 inhibitor (prexasertib).

**Figure 10:**
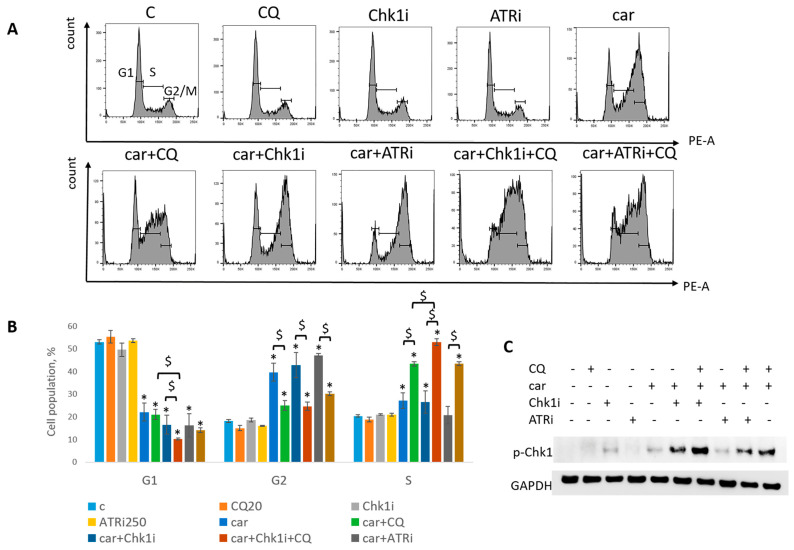
CQ potentiated the chemotherapeutic effect of carboplatin and RSR inhibitors in SKBR3 cells. (**A**) Cell cycle analysis by flow cytometry. Cells were treated with 50 µM carboplatin + ATRi (0.25 µM)/Chk1i (1 nM) and combination of carboplatin + ATRi/Chk1i + CQ (20 µM). The representative images with cell cycle phase distribution are shown for each experimental group. (**B**) Histogram represents the percentage of cells in G1, S and G2/M cell cycle phases. The averages ± st. dev. are shown. * *p* < 0.01 compared with non-treated cells; ^$^ *p* < 0.01. (**C**) Western blot analysis of replication stress marker p-Chk1 (Ser345). GAPDH used as a loading control. Experiments were repeated three times. C—control (non-treated cells), CQ—chloroquine, car—carboplatin, ATRi—ATR inhibitor (ceralasertib), Chk1i—Chk1 inhibitor (prexasertib).

The most known RSR inhibitors are ATR and Chk1 inhibitors (ATRi/Chk1i) [31]. Tumors treatment with combination of ATRi/Chk1i and chemotherapeutic drugs has good synergistic effect and these inhibitors are under clinical investigation. However, adverse effects were observed. We hypothesized that supplementation of the above combinations with CQ would be more effective at tumor killing and would allow to reduce the concentration of ATRi and Chk1i. As we expected to find the synergism between ATRi/Chk1i and CQ, we selected the concentration that would allow some tumor cells to resume their proliferation post treatment when used in combination with carboplatin separately from each other (carboplatin + ATRi/Chk1i and carboplatin + CQ) (Figure 7 and Figure 8). Combinations of three drugs (carboplatin + ATRi + CQ and carboplatin + Chk1i + CQ) completely abrogated the ability of tumor cells to re-proliferate post treatment. We confirmed using breast cancer cell lines that both carboplatin and combination of CQ + carboplatin caused S-phase cell cycle arrest with more pronounced increase in carboplatin + CQ-treated groups. ATRi/Chk1i in combination with carboplatin led to G1 phase decrease in both cell lines and increase in the number of cells in G2/M phase in SKBR3 cells. Three agents’ combination treatment also resulted in significant S phase induction compared to experimental groups without CQ. Moreover, we observed the G2/M phase decrease in SKBR3 cells in the presence of CQ in combination treatment and also in MCF7 cells when combination of carboplatin + ATRi + CQ was applied (compared with carboplatin + ATRi experimental group). Note that in SKBR3 cells, Chk1 inhibitor and CQ had synergistic effect on S phase increase (Figure 9A,B and Figure 10A,B).

Analysis of replication stress marker, p-Chk1 (Ser345), revealed its significant increase when Chk1i was applied both as single agent and in combinations treatment. The result is in accordance with others [32,33]. Supplementation with CQ increased the level of p-Chk1 in three drugs combination-treated group (ATRi/Chk1i + carboplatin + CQ) in MCF7 and SKBR3 cells (Figure 9C and Figure 10C). The results reflected the cell cycle results—increase in S (or G1) phase was accompanied by the increase in the level of p-Chk1 when CQ was applied. Chk1 phosphorylation at serine 345 served as a biochemical marker of Chk1 inhibition and was observed in other studies [32,34].

Taken together, we showed that CQ potentiated the chemotherapeutic effect of the combinations of carboplatin and ATRi/Chk1i.

**Figure 11:**
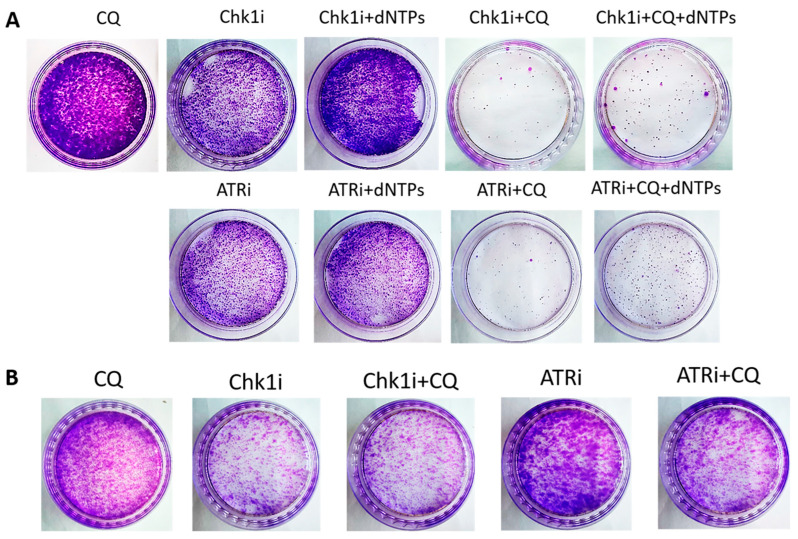
The ability of MCF7 (**A**) and SKBR3 (**B**) cells to re-proliferate after drugs (ATRi/Chk1i + CQ) treatment. Cells were exposed to ATRi (8 µM)/Chk1i (20 nM (MCF7); 4 nM (SKBR3)) and to the combination of ATRi/Chk1i + CQ (15 µM (MCF7); 40 µM (SKBR3)) for 24 h; next day drug-free medium was replenished, cells were re-seeded at low density and allowed to form colonies which were stained with crystal violet 10 days after. The experiments were repeated at least three times; the representative experiments are shown. CQ—chloroquine, ATRi—ATR inhibitor (ceralasertib), Chk1i—Chk1 inhibitor (prexasertib), dNTPs—deoxyribonucleotides.

**Figure 12:**
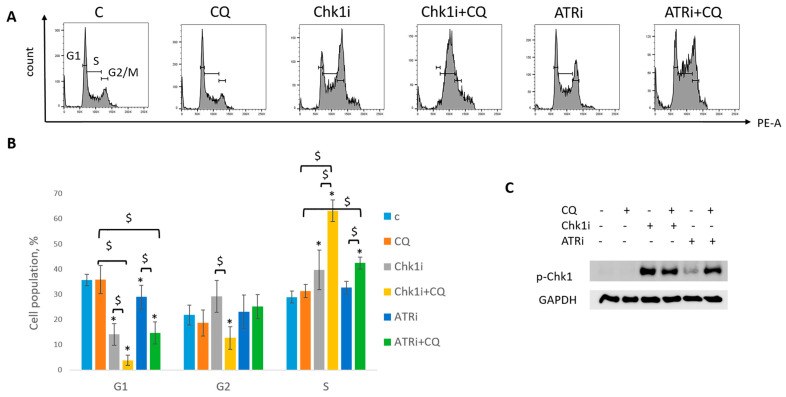
CQ potentiated the chemotherapeutic effect of RSR inhibitors in MCF7 cells. (**A**) Cell cycle analysis by flow cytometry. Cells were treated with ATRi (0.25 µM)/Chk1i (20 nM) and combination ATRi/Chk1i + CQ (15 µM). The representative images with cell cycle phase distribution are shown for each experimental group. (**B**) Histogram represents the percentage of cells in G1, S and G2/M cell cycle phases. The averages ± st. dev. are shown. * *p* < 0.01 compared with non-treated cells; ^$^ *p* < 0.01. (**C**) Western blot analysis of replication stress marker p-Chk1 (Ser345). GAPDH used as a loading control. The experiments were repeated three times. C—control (non-treated cells), CQ—chloroquine, ATRi—ATR inhibitor (ceralasertib), Chk1i—Chk1 inhibitor (prexasertib).

**Figure 13:**
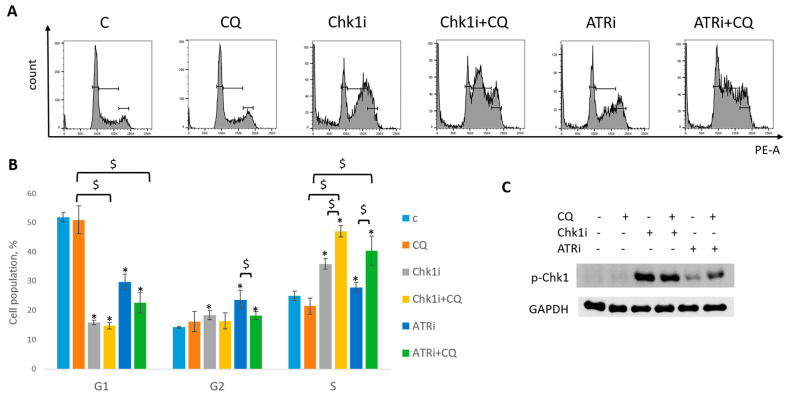
CQ potentiated the chemotherapeutic effect of RSR inhibitors in SKBR3 cells. (**A**) Cell cycle analysis by flow cytometry. Cells were treated with ATRi (0.25 µM)/Chk1i (4 nM) and combination ATRi/Chk1i + CQ (40 µM). The representative images with cell cycle phase distribution are shown for each experimental group. (**B**) Histogram represents the percentage of cells in G1, S and G2/M cell cycle phases. The averages ± st. dev. are shown. * *p* < 0.01 compared with non-treated cells; ^$^ *p* < 0.01. (**C**) Western blot analysis of replication stress marker p-Chk1 (Ser345). GAPDH used as a loading control. The experiments were repeated three times. C—control (non-treated cells), CQ—chloroquine, ATRi—ATR inhibitor (ceralasertib), Chk1i—Chk1 inhibitor (prexasertib).

Next, we asked if CQ could potentiate the effect of RSR inhibitors without carboplatin, as these drugs are also used as monotherapy treatment. Concentration of inhibitors when acting as single agents were selected as previously described—to allow some cells to resume their proliferation post treatment (Figure 11). Concentration of CQ was the same as with carboplatin for each cell line. As expected, supplementation with CQ significantly inhibited the ability of MCF7 cells to re-proliferate post treatment (Figure 11A). The effect was cell-type-dependent—MCF7 cells showed complete inhibition of cell proliferation resumption, while SKBR3 cells demonstrated only modest decrease in re-proliferating potential when applied with ATRi (Figure 11B). However, CQ significantly increased cell cycle S phase in both cell lines (Figure 12A,B and Figure 13A,B). The level of replication stress marker—p-Chk1 (Ser 345)—was significantly increased when CQ was used together with ATRi; Chk1i showed strong induction of p-Chk1 by itself, with no further increase when used in combination with CQ (Figure 12C and Figure 13C).

Taken together, we demonstrated that the effect of CQ added to RSR inhibitors used as single agents for tumor treatment was not so clear—despite the induction of S phase and p-Chk1 level indicating the replication stress increase, the influence of the combination on cell proliferation resumption is uncertain as it was completely abrogated in MCF7 cells, with no significant effect in SKBR3 cells.

The rescue experiment demonstrated that nucleotide supplementation reversed the described effects of ATRi/Chk1i and CQ in MCF7 cells. We found the increase in the number of cells able to re-proliferate after drugs treatment compared to the experimental groups without dNTPs (Figure 7 and Figure 11). At the same time, we could not detect phenotype reversion in SKBR3 cells (Figure 8).

## 3. Discussion

Monotherapy with genotoxic drugs used for cancer treatment has some unfavorable outcomes such as serious side effects, drug resistance development, etc. They can be decreased by reducing the drug dose but, at the same time, the efficiency of therapy is also decreased, resulting in insufficient tumor killing and tumor reoccurrence. The solution of this problem is to treat cancer with combination chemotherapy when different agents target different pathways, act synergistically and thus reduce the drug toxicity [31].

This study demonstrated the synergism between lysosomal inhibitors (CQ and NH_4_Cl) and carboplatin in tumor cells. The combination suppressed the ability of cells to resume proliferation post drug treatment. Carboplatin monotherapy is characterized by the development of toxicities such as thrombocytopenia, neutropenia, and anaemia [35]. Our data suggested that applying CQ as an adjuvant to carboplatin would reduce its toxicity because of decreasing the effective dose of carboplatin. Moreover, as CQ has some overlapping toxicities with carboplatin, such as gastrointestinal, it seems important that low doses of combination chemotherapy can be applied to achieve the treatment efficiency, thus minimizing the possible side effects. The potential mechanism of CQ action is its ability to enhance the replication stress caused by carboplatin partly by decreasing the amount of nucleotides needed for DNA synthesis.

The effect of combination treatment with CQ and chemotherapeutic drugs were evaluated in previous studies [17,36], and the synergism between these agents was also shown. However, none indicated the involvement of CQ in targeting the replication stress pathways. One study pointed out that the effect of combination of CQ and cisplatin treatment in EOC cells was similar to the combination of ATRi and cisplatin [37]. Here, we confirmed and explained this observation. CQ, as well as ATRi, targets the RSR pathway, leading to the increase in replication stress markers—p-Chk1 (Ser345) and S phase of cell cycle that eventually resulted in increased cell death.

A group of scientists previously demonstrated that CQ was able to inhibit de novo nucleotides’ biosynthesis, which could be the reason for the increased replication stress and its combination with VE-822 (ATR inhibitor) synergistically inhibiting PDAC cell growth [23]. Our study also showed the synergism between CQ and ATRi in MCF7 cells and, moreover, found that CQ effect was not restricted to RSR inhibitors (ATRi and Chk1i) but was presented in combination with platinum drugs (carboplatin and cisplatin). The rescue experiments aimed to determine if nucleotide presence in culture medium would reverse the effect of CQ in combination with the described agents demonstrated the increased number of MCF7 cells able to resume their proliferation post treatment compared to the group without exogenous dNTPs. It indicated that, at least partly, a cell’s phenotype caused by CQ is due to its ability to inhibit de novo nucleotide synthesis.

The rescue experiment in SKBR3 cells was successful only when CQ was used together with carboplatin. However, when the combination of carboplatin + RSRi + CQ was used, we could not reverse the effect by nucleotide supplementation. This was probably because the more severe stress was induced.

Our study demonstrated that CQ in combination with different agents (carboplatin, cisplatin, RSR inhibitors) caused an increase in S (or G1) phase induction, which correlated with significant increase in Chk1 phosphorylation at serine 345 (common marker of replication stress). As a result, tumor cells completely lost the ability to resume their proliferation post treatment. SKBR3 cells demonstrated stronger S phase increase in combination-treated group compared to single carboplatin treatment than MCF7 cells. The same was true when three drugs combinations were applied (carboplatin + ATRi/Chk1i + CQ)—S phase induction was more pronounced in SKBR3 cells than in MCF7 in the presence of CQ. Here, CQ also demonstrated the synergism in the induction of Chk1 phosphorylation at serine 345 with carboplatin and ATRi/Chk1i, with more pronounced effect in carboplatin + ATRi + CQ groups (compared to the groups without CQ). The long-term result of CQ supplementation was similar to carboplatin + CQ combination treatment—significant reduction in the number of tumor cells able to resume their proliferation post treatment. This study demonstrated a successful example of multiple-low-dose therapy (MLD) [38]—a perspective strategy for cancer treatment as it allows us to overcome single drug treatment resistance and to reduce the toxicity. The effect of CQ supplementation on single RSR inhibitors (without genotoxic drug) was controversial. MCF7 cells demonstrated results similar to those described above, but in SKBR3 cells, despite the significant induction of replication stress (increase in S phase and p-Chk1 level), the reduction in cells number able to re-proliferate post treatment was not observed. As was shown earlier, SKBR3 cell line is more sensitive to Chk1 inhibition than MCF7 [39]. We propose that there is a threshold of DNA damage caused by RSRi—above it, sensitive cells respond to the inhibitors by rapid cell death, whereas resistant have a prolonged response; during this time, they can repair the DNA. Below the threshold, when the DNA damage is not critical, cells successfully proliferate after finishing the DNA repair process. So CQ may promote increased sensitivity of resistant cells by enhancing the replication stress (through reducing the nucleotide biosynthesis). A similar result was obtained in another study—CQ potentiated the effect of Chk1 inhibitor on cell viability in HT29 cells (resistant to Chk1i [39]) and did not increase the Chk1i effect on cell death in U2OS cells (sensitive to Chk1i [39]) [40].

Based on our results (Western blots in Figure 9, Figure 10, Figure 12 and Figure 13), we propose that CQ induced Chk1 phosphorylation in ATR-independent as its administration together with ATRi significantly enhanced Chk1 phosphorylation compared to the experimental groups without CQ. At the same time, when CQ was applied together with Chk1i, we could not detect further increase in phospho-Chk1 protein level. The increase in Chk1 phosphorylation in three-agents-treated groups (carboplatin + Chk1i + CQ) compared to groups without CQ could be explained by the use of low Chk1i concentration.

In recent years, the RSR inhibitors have been intensively developed and tested in preclinical and clinical studies [41,42] both as single agents and in combination with genotoxic drugs. But, most of them have serious adverse effects caused by high dosage needed to reach the therapeutic effect. Prexasertib demonstrated good antitumor efficiency both as monotherapy and combination chemotherapy [43] but in phase II trials the toxicity was observed. It indicated the necessity to find a way to reduce prexasertib dosage without losing its therapy efficacy. Ceralasertib faced the same problems. Our results demonstrated that supplementation of low-dose combination of carboplatin + ATRi/Chk1i with low-dose CQ could significantly increase the therapeutic effect. In the same way, the effective dose reduction and the decrease in cells’ ability to re-proliferate post treatment would be achieved by CQ introduction in therapeutic treatment with ATRi/Chk1i as single agents at least in some tumors.

As CQ increased the replication stress, we can predict that it would exhibit the synergism with the agents that targeted RSR pathways, including nucleoside analogs (gemcitabine, 5-fluorouracil), alkylating agents (cyclophosphamide, dacarbazine, temozolomide), topoisomerase I and II (doxorubicin, etoposide), PARP and used in this study—ATRi and Chk1i [26]. Indeed, some studies have already demonstrated the ability of CQ to potentiate the effect of the above agents [17]—doxorubicin [44,45], temozolomide [46], gemcitabine [22], while others remained to be investigated.

## 4. Materials and Methods

### 4.1. Chemical Reagents

Carboplatin and cisplatin were purchased from United Biotech (New Delhi, India). CQ purchased from Sigma (St. Louis, MO, USA) was suspended in distilled water (stock concentration 50 mM). ATR inhibitor, ceralasertib, was purchased from Sigma (St. Louis, MO, USA) and suspended in DMSO (stock concentration 10 mM). Chk1 inhibitor, prexasertib, purchased from Macklin (Shanghai, China) was suspended in DMSO (stock concentration 10 mM). NH4Cl was purchased from Applichem (Darmstadt, Germany) and suspended in distilled water (stock concentration 1 M). dNTPs were purchased from Evrogen (Moscow, Russia) and used at final concentration 20 µM [30].

### 4.2. Cell Lines

All cell lines used in the study were obtained from the collection of N.N. Blokhin National Medical Research Center of Oncology of the Ministry of Health of the Russian Federation (Moscow, Russia). They were cultured in DMEM (Paneco, Moscow, Russia) supplemented with 10% FBS (Biosera, Cholet, France) and a mix of antibiotics (penicillin/streptomycin) (Paneco, Moscow, Russia) and were grown at 37 °C in a 5% CO_2_ incubator.

### 4.3. Cell Growth Assay

Cells (MCF7 3 × 10^4^, SKBR3 7 × 10^3^, HCT116 4 × 10^4^) were seeded at 6-well plates (Nest, Wuxi, China). A total of 24 h after, the drugs were applied. At 24 h post drugs treatment, the new drug-free medium was replenished. Cells were stained with trypan blue and only live cells were counted using hemocytometer (Paneco, Moscow, Russia).

### 4.4. MTT

3 × 10^3^ cells (MCF7, SKBR3, HCT116) were seeded at 96-well plates (Nest, Wuxi, China). A total of 24 h after, carboplatin was applied. At 24 h post drug treatment, the new drug-free medium was replenished. At 72 h post treatment, the reagent MTT ((3-(4,5-Dimethylthiazol-2-yl)-2,5-Diphenyltetrazolium Bromide)) (Sigma, St. Louis, MO, USA) (0.5 mg/mL) was added to the cells in culture medium. Cells were incubated with reagent for 2 h in CO_2_ incubator. The medium was removed and cells were suspended in 100 µL of DMSO (Paneco, Moscow, Russia). The absorbance of each well was measured at a wavelength of 540 nm using a plate reader TECAN (Grödig, Austria).

### 4.5. Cells Re-Proliferation Analysis

9 × 10^4^ cells (MCF7, SKBR3, HCT116) were seeded at 6-well plates (Nest, Wuxi, China). A total of 24 h after, carboplatin was applied. At 24 h post drug treatment, the new drug-free medium was replenished. Then, cells were re-seeded at low density (one third of initial seeding density) at 6 cm plates and cultured for 10 days until the colonies could be visualized. The medium was replenished every three days. Then, the cells were fixed with 4% paraformaldehyde (Sigma, St. Louis, MO, USA) for 10 min followed by staining with crystal violet (GoldBio, St. Louis, MO, USA) for 20 min.

### 4.6. Flow Cytometry

#### 4.6.1. Apoptosis Assay

Apoptosis was detected using annexin V/PI staining (BD Bioscience, San Jose, CA, USA). 9 × 10^4^ cells (MCF7, SKBR3, HCT116) were seeded at 6-well plates (Nest, Wuxi, China). A total of 24 h after, carboplatin was applied. At 24 h post drug treatment, the new drug-free medium was replenished. At indicated time points, the cells were collected and suspended in 100 µL of 1× binding buffer. Then, cells were incubated with 5 µL of APC-conjugated annexin-V and c of PI for 15 min at room temperature in darkness. After two washes of antibodies, cells were analyzed using flow cytometry (FACS Canto II, BD Bioscience, San Jose, CA, USA). The results were processed using FlowJo software, version 10.8.1.

#### 4.6.2. Cell Cycle Analysis

Cells were seeded and drugs were applied as described for apoptosis assay. At indicated time points, the cells were collected and suspended in cell cycle buffer (0.1% sodium citrate, 0.3% NP-40, 50 µg/mL PI, and 50 µg/mL RNase A). After 30 min of incubation in darkness, cell cycle distribution was analyzed using flow cytometry (FACS Canto II, BD Bioscience, San Jose, CA, USA). The results were processed using FlowJo software.

#### 4.6.3. Western Blot Analysis

Cells were lysed in RIPA buffer (50 mM Tris-HCl (pH 7.4–8.0), 150 mM NaCl, 1% NP-40, 0.1% SDS, and 0.5% sodium deoxycholate). Protein concentration was determined using Bradford method (BioRad, Hercules, CA, USA). In total, 30–40 µg of proteins were loaded in each well for SDS-PAGE electrophoresis (10–15% resolving gels were used) (BioRad system, Hercules, CA, USA). Proteins from the gel were wet transferred into the PVDF membrane (BioRad, Hercules, CA, USA). After transfer was completed, the membrane was blocked in 5% non-fat dry milk (BioRad, Hercules, CA, USA) or 3% bovine serum albumin (Sigma, St. Louis, MO, USA). The incubation with primary antibodies was performed overnight at +4 °C. After washing the membrane in PBS-T solution it was treated with secondary antibodies conjugated with HRP (anti-rabbit (Cell Signaling, Danvers, MA, USA) and anti-mouse (Abclonal, Woburn, MA, USA)) for 40 min at room temperature. Proteins were visualized with Clarity Western ECL Substrate (Bio-Rad, Hercules, CA, USA) using ImageQuant LAS 4000 (GE Healthcare, Chicago, IL, USA). The following primary antibodies were used: p-Chk1 (Ser345), LC3A/B (Cell Signaling, Danvers, MA, USA), GAPDH (Elk Biotechnology, Wuhan, China).

### 4.7. Statistics

The statistical analysis was performed using Mann–Whitney test. The Benjamini–Hochberg correction was applied for multiple comparisons. Data analysis was performed using FlowJo and GraphPad Prism software version 8.0.2. *p* value ˂ 0.05 was considered significant.

## Data Availability

The original contributions presented in this study are included in the article and Supplementary Materials. Further inquiries can be directed to the corresponding author.

## Supplementary Materials

The following supporting information can be downloaded at: https://www.mdpi.com/article/10.3390/ijms27020856/s1.
