# Supplementary material for: Chloroquine Potentiates the Chemotherapeutic Effect of Carboplatin and ATR/Chk1 Inhibitors by Increasing the Replication Stress
Source: Int J Mol Sci. 2026 Jan 15;27(2):856. doi: 10.3390/ijms27020856 (PMC12840919; doi:10.3390/ijms27020856)
Supplement: Supplementary file 1 [file ijms-27-00856-s001.zip › ijms-4041585-supplementary.pdf]

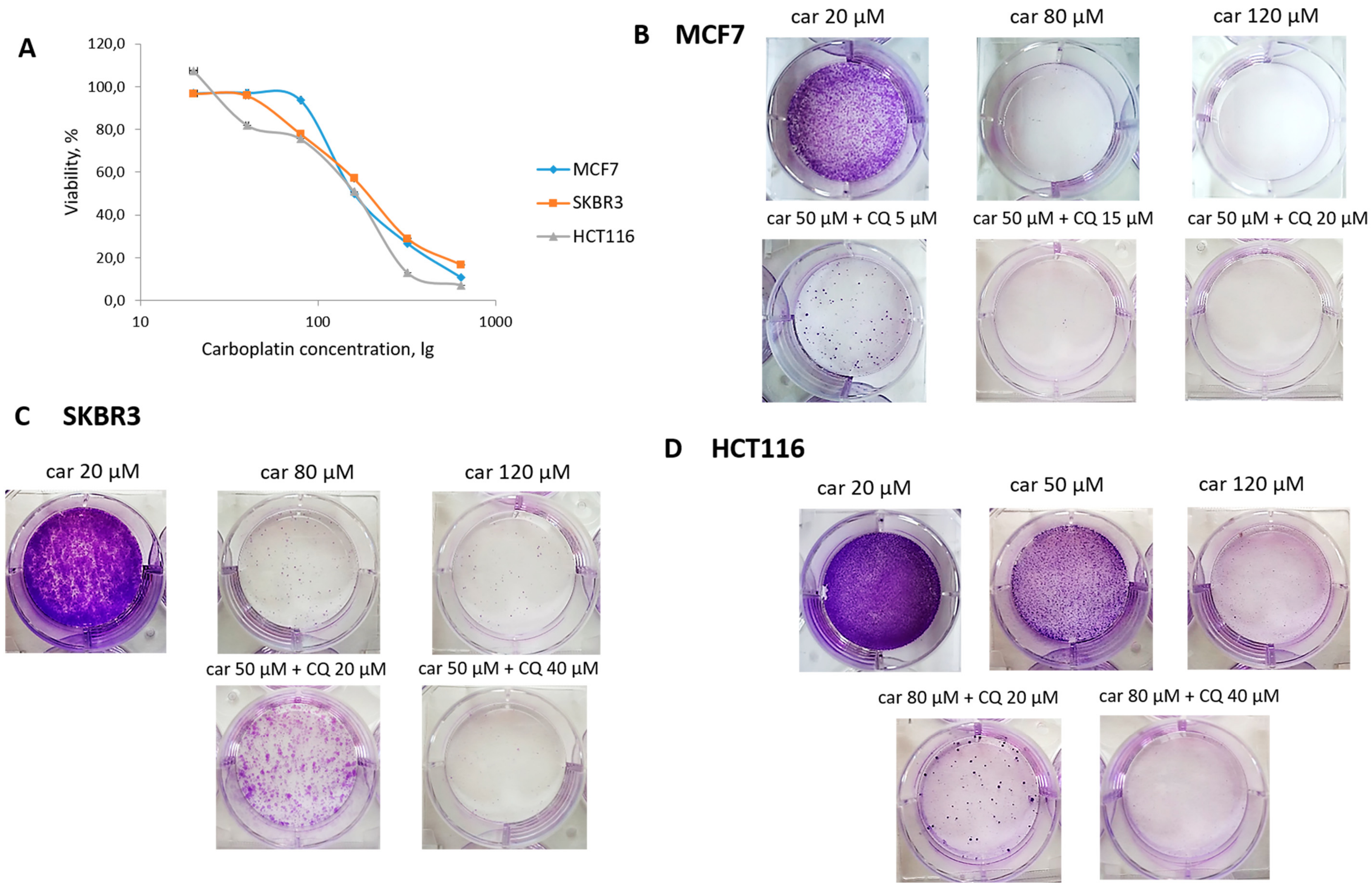
A
120,0
100,0
80,0
60,0
40,0
20,0
0,0
Viability, %
10
100
1000
Carboplatin concentration, lg
MCF7
SKBR3
HCT116
B MCF7
car 20 μM
car 80 μM
car 120 μM
car 50 μM + CQ 5 μM
car 50 μM + CQ 15 μM
car 50 μM + CQ 20 μM
C SKBR3
car 20 μM
car 80 μM
car 120 μM
car 50 μM + CQ 20 μM
car 50 μM + CQ 40 μM
D HCT116
car 20 μM
car 50 μM
car 120 μM
car 80 μM + CQ 20 μM
car 80 μM + CQ 40 μM

Figure S1. Determination of drugs concentration. (A) MTT assay. Three tested cell lines (MCF7, SKBR3, HCT116) were exposed to a range of carboplatin concentrations (20 μM – 640 μM) for 24 h. OD600 were determined 72 h after drug treatment. (B - D) The ability of cells (MCF7 (B), SKBR3 (C) and HCT116 (D)) to re-proliferate after drugs treatment was determined by crystal violet staining. Cells were re-seeded at low density 24 h after the drugs were applied and allowed to form colonies. The experiments were repeated at least two times; the representative experiments are shown. C – control (non-treated cells), CQ – chloroquine, car – carboplatin.

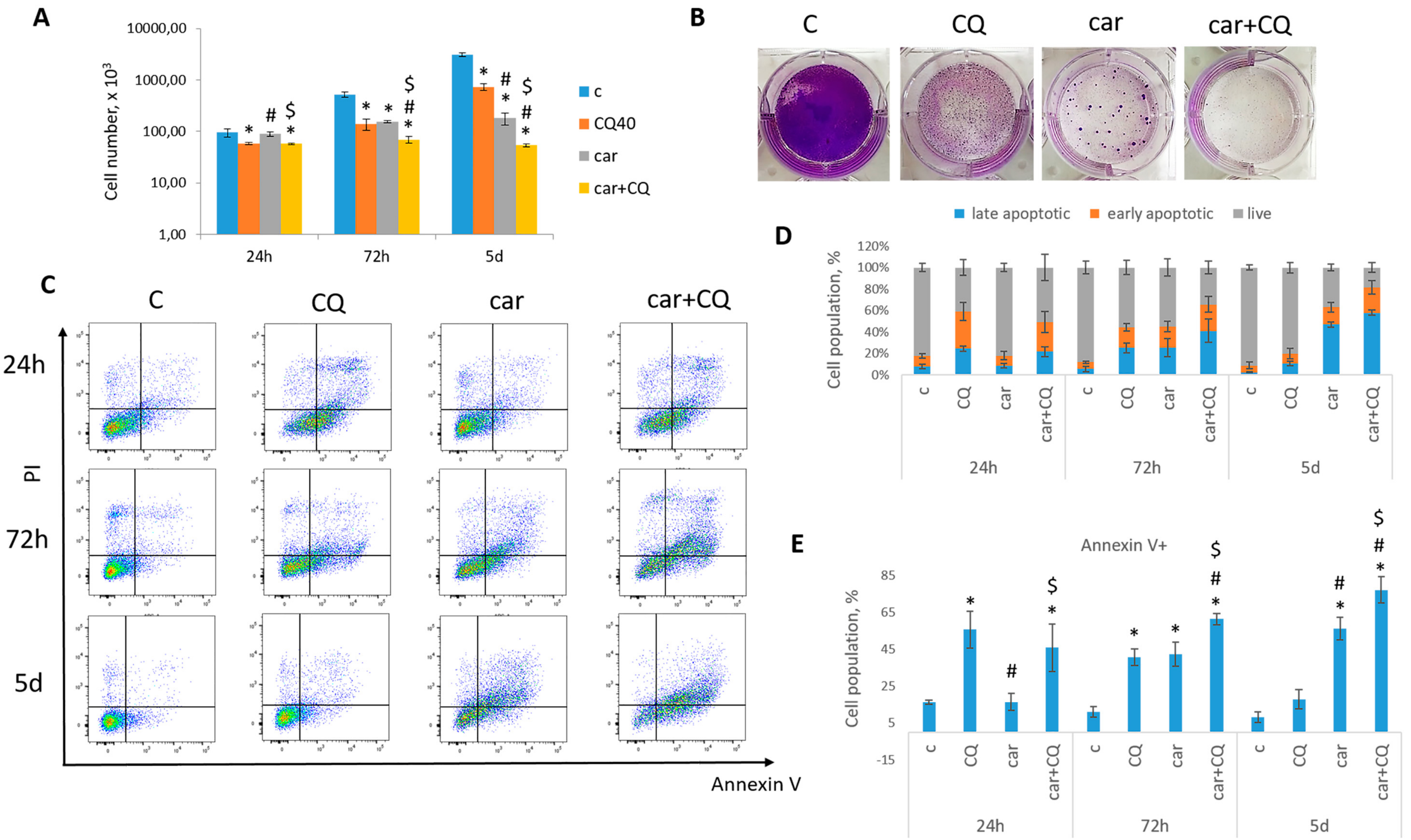

A
Cell number, x 10^3
10000,00
1000,00
100,00
10,00
1,00
24h
72h
5d
c
CQ40
car
car+CQ
B
C
CQ
car
car+CQ
C
C
CQ
car
car+CQ
24h
72h
5d
PI
Annexin V
D
late apoptotic
early apoptotic
live
Cell population, %
120%
100%
80%
60%
40%
20%
0%
c
CQ
car
car+CQ
24h
72h
5d
E
Annexin V+
Cell population, %
85
65
45
25
5
-15
c
CQ
car
car+CQ
24h
72h
5d

Figure S2. CQ potentiated the chemotherapeutic effect of carboplatin in HCT116 cells. (A) Cell growth assay. $4x10^4$ HCT116 cells were seeded at day 0. Drugs were applied at day 1; next day drug-free media were replenished (24 h). The cells were counted in three time points (24 h, 72 h and 5 d). The experiments were repeated in two replicates three times. The averages ± st. dev. are shown. (B) The ability of HCT116 cells to re-proliferate after drugs treatment. Cells were exposed to carboplatin and to the combination of carboplatin and CQ for 24 h; next day drug-free media were replenished, cells were re-seeded at low density and allowed to form colonies which were stained with crystal violet 10 days after. The experiments were repeated at least three times; the representative experiments are shown. (C - D) Cells were treated with carboplatin and with the combination of carboplatin and CQ for 24 h; next day drug-free media were replenished. Annexin V/PI staining was performed in three time points (24 h, 72 h and 5 d). (C) The representative flow cytometry images are shown for each time point. (D) Histogram demonstrated the percentage of live, early and late apoptotic cells in each experimental group. (E) Cell populations stained positive for annexin V are shown. Experiments were repeated at least three times. The averages ± st. dev. are shown. * $p < 0{,}01$ compared with non-treated cells; # $p < 0{,}01$ compared to CQ treated cells; $ $p < 0{,}01$ two drug treated cells compared with single drug (carboplatin) treated cells. C – control (non-treated cells), CQ – chloroquine, car – carboplatin.

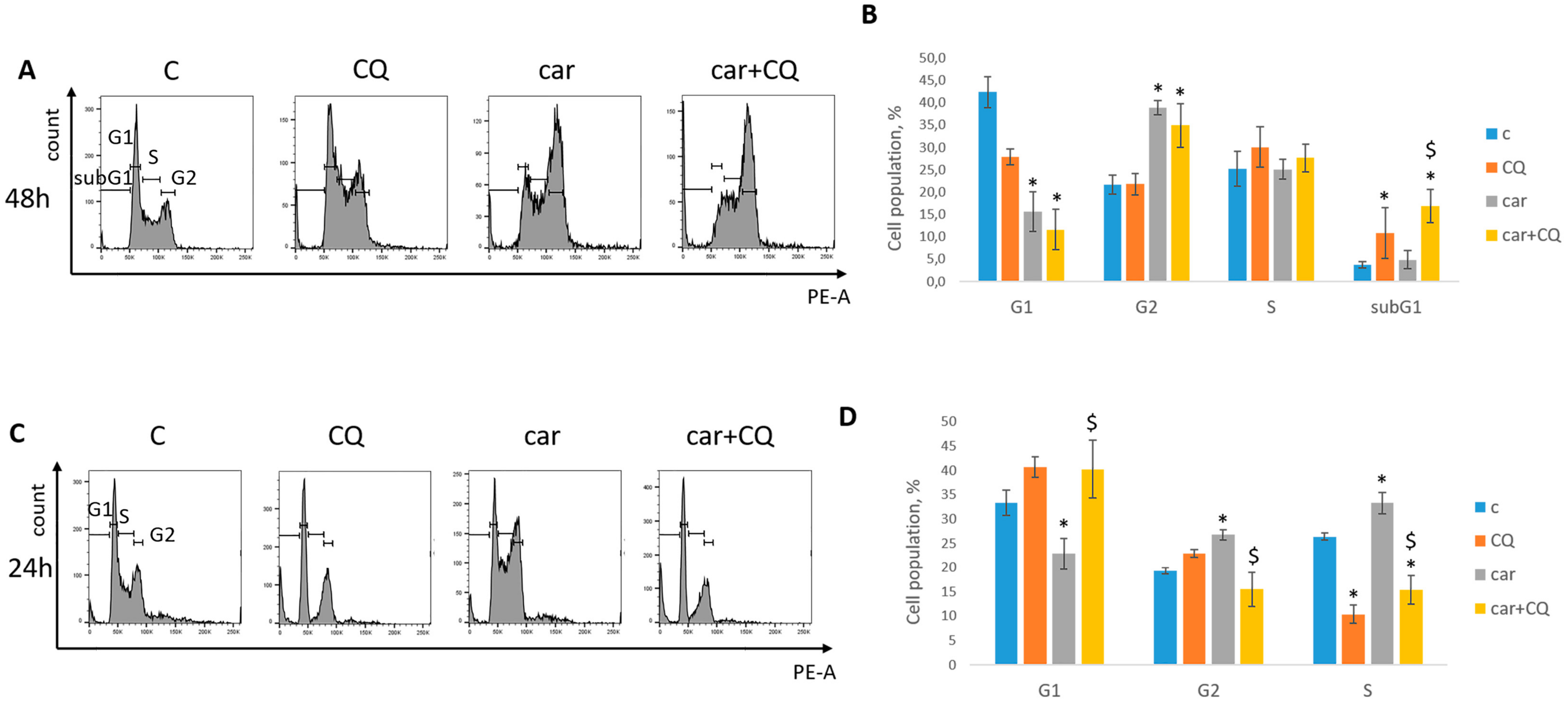

Figure S3. Cell cycle analysis of MCF7 and HCT116 cells. Cells were treated with carboplatin and combination of carboplatin and CQ. The representative images with cell cycle phase distribution are shown for each experimental group 48 h post treatment for MCF7 (A) and 24 h post treatment for HCT116 (C) cells. (B) Histogram represents the percentage of MCF7 cells in G1, S, G2/M and subG1 cell cycle phases. (D) Histogram represents the percentage of HCT116 cells in G1, S and G2/M cell cycle phases. Experiments were repeated at least three times. The averages ± st. dev. are shown. * $p < 0,01$ compared with non-treated cells; $ $p < 0,01$ two drug treated cells compared with single drug (carboplatin) treated cells.

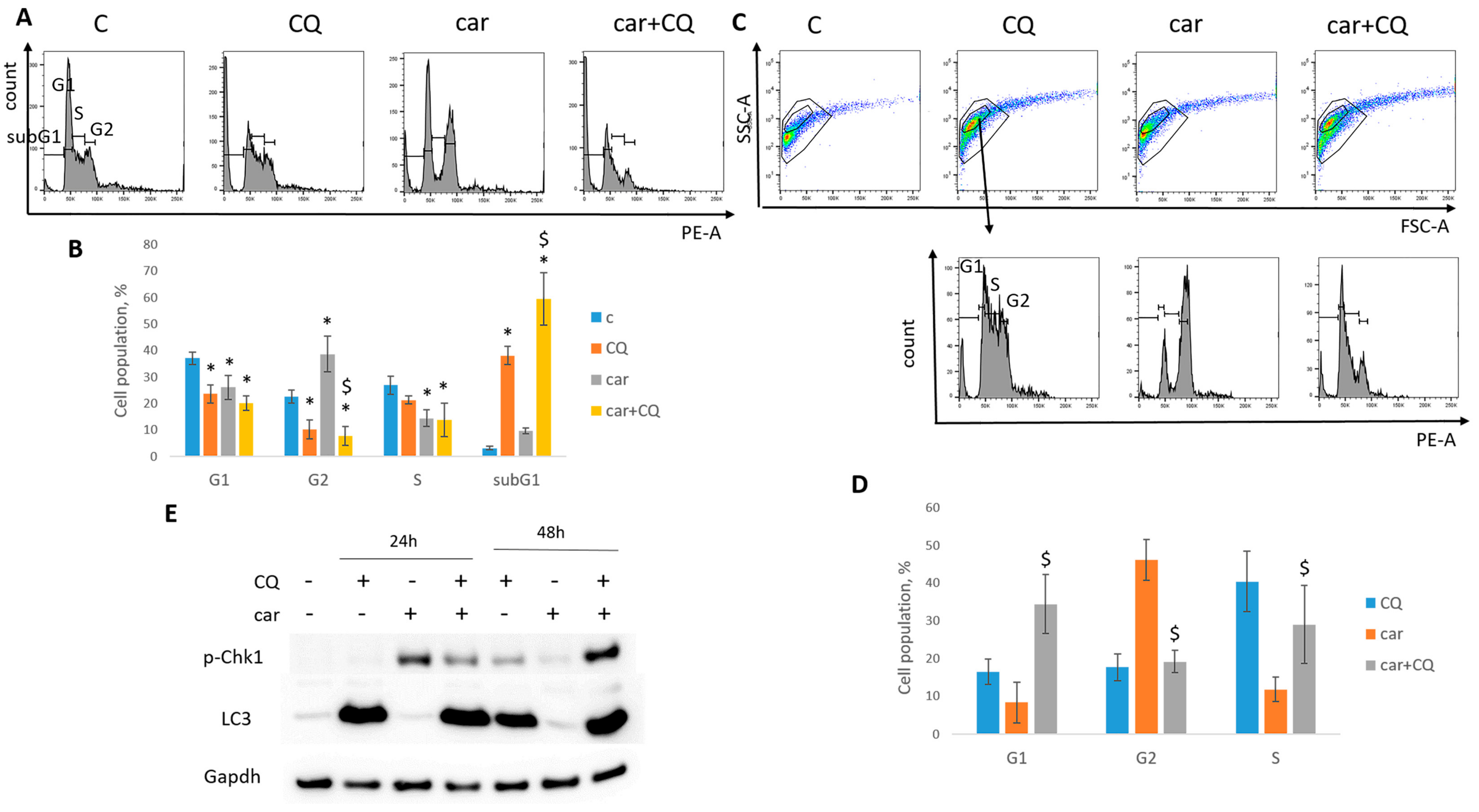
A
C
CQ
car
car+CQ
count
G1
S
G2
subG1
PE-A
B
Cell population, %
80
70
60
50
40
30
20
10
0
G1
G2
S
subG1
c
CQ
car
car+CQ
*
$
C
C
CQ
car
car+CQ
SSC-A
FSC-A
count
G1
S
G2
PE-A
D
Cell population, %
60
50
40
30
20
10
0
G1
G2
S
CQ
car
car+CQ
$
E
24h
48h
CQ - + - + + - +
car - - + + - + +
p-Chk1
LC3
Gapdh

Figure S4. CQ enhanced replication stress in HCT116 cells. (A) Cell cycle analysis by flow cytometry. Cells were treated with carboplatin and combination of carboplatin and CQ. The representative images with cell cycle phase distribution are shown for each experimental group. (B) Histogram represents the percentage of cells in G1, S and G2/M cell cycle phases. (C) Strategy for gating the live cell population with larger size compared to control cells (up). The representative images with cell cycle phases distribution in this population are shown for each experimental group (bottom). (D) Histogram represents the percentage of cells in G1, S and G2/M cell cycle phases. Experiments were repeated at least three times. The averages ± st. dev. are shown. * $p < 0{,}01$ compared with non-treated cells; $ $p < 0{,}01$ two drug treated cells compared with single drug (carboplatin) treated cells. (E) Western blot analysis of replication stress marker p-Chk1 (Ser345) and autophagy marker LC3A/B. GAPDH used as a loading control. C – control (non-treated cells), CQ – chloroquine, car – carboplatin.

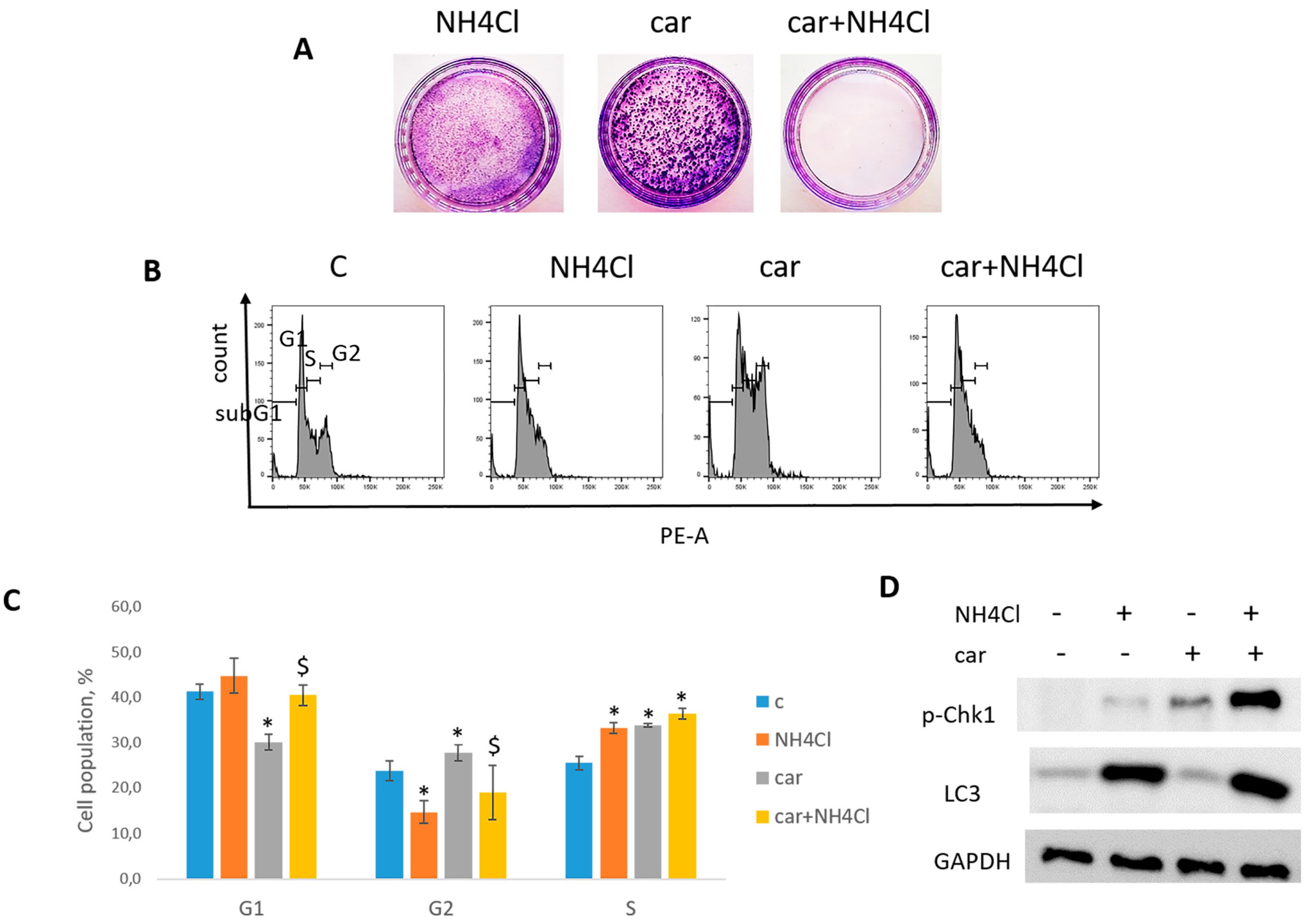
A
NH4Cl
car
car+NH4Cl
B
C
NH4Cl
car
car+NH4Cl
count
G1
S
G2
subG1
PE-A
C
Cell population, %
60,0
50,0
40,0
30,0
20,0
10,0
0,0
G1
G2
S
c
NH4Cl
car
car+NH4Cl
D
NH4Cl - + - +
car - - + +
p-Chk1
LC3
GAPDH

Figure S5. Lysosomal inhibition with NH_4Cl leaded to enhanced replication stress and inhibition of MCF7 cells viability. Cells were treated with carboplatin and with the combination of carboplatin and NH_4Cl for 24 h; next day drug-free media were replenished. A) Cells were re-seeded at low density and allowed to form colonies which were stained with crystal violet 10 days after. The experiments were repeated at least three times; the representative experiments are shown. B) The representative images with cell cycle phases distribution 24 h post treatment are shown for each experimental group. C) Histogram represents the percentage of cells in G1, S and G2/M cell cycle phases. Experiments were repeated at least three times. The averages ± st. dev. are shown. * $p < 0{,}01$ compared with non-treated cells; $ $p < 0{,}01$ two drug treated cells compared with single drug (carboplatin) treated cells. D) Western blot analysis of replication stress marker p-Chk1 (Ser345) and autophagy marker LC3A/B. GAPDH used as a loading control. C – control (non-treated cells), car – carboplatin.
